# Sequestration of free cholesterol in cell membranes by prions correlates with cytoplasmic phospholipase A_2 _activation

**DOI:** 10.1186/1741-7007-6-8

**Published:** 2008-02-12

**Authors:** Clive Bate, Mourad Tayebi, Alun Williams

**Affiliations:** 1 Department of Pathology and Infectious Diseases, Royal Veterinary College, Hawkshead Lane, North Mymms, Herts, AL9 7TA, UK

## Abstract

**Background:**

The transmissible spongiform encephalopathies (TSEs), otherwise known as the prion diseases, occur following the conversion of the normal cellular prion protein (PrP^C^) to an alternatively folded isoform (PrP^Sc^). The accumulation of PrP^Sc ^within the brain leads to neurodegeneration through an unidentified mechanism. Since many neurodegenerative disorders including prion, Parkinson's and Alzheimer's diseases may be modified by cholesterol synthesis inhibitors, the effects of prion infection on the cholesterol balance within neuronal cells were examined.

**Results:**

We report the novel observation that prion infection altered the membrane composition and significantly increased total cholesterol levels in two neuronal cell lines (ScGT1 and ScN2a cells). There was a significant correlation between the concentration of free cholesterol in ScGT1 cells and the amounts of PrP^Sc^. This increase was entirely a result of increased amounts of free cholesterol, as prion infection reduced the amounts of cholesterol esters in cells. These effects were reproduced in primary cortical neurons by the addition of partially purified PrP^Sc^, but not by PrP^C^. Crucially, the effects of prion infection were not a result of increased cholesterol synthesis. Stimulating cholesterol synthesis via the addition of mevalonate, or adding exogenous cholesterol, had the opposite effect to prion infection on the cholesterol balance. It did not affect the amounts of free cholesterol within neurons; rather, it significantly increased the amounts of cholesterol esters. Immunoprecipitation studies have shown that cytoplasmic phospholipase A_2 _(cPLA_2_) co-precipitated with PrP^Sc ^in ScGT1 cells. Furthermore, prion infection greatly increased both the phosphorylation of cPLA_2 _and prostaglandin E_2 _production.

**Conclusion:**

Prion infection, or the addition of PrP^Sc^, increased the free cholesterol content of cells, a process that could not be replicated by the stimulation of cholesterol synthesis. The presence of PrP^Sc ^increased solubilisation of free cholesterol in cell membranes and affected their function. It increased activation of the PLA_2 _pathway, previously implicated in PrP^Sc ^formation and in PrP^Sc^-mediated neurotoxicity. These observations suggest that the neuropathogenesis of prion diseases results from PrP^Sc ^altering cholesterol-sensitive processes. Furthermore, they raise the possibility that disturbances in membrane cholesterol are major triggering events in neurodegenerative diseases.

## Background

Cholesterol levels within the brain may affect the pathogenesis of some neurodegenerative diseases including Alzheimer's and Parkinson's diseases and multiple sclerosis [1,2]. Neuronal cholesterol levels are also thought to affect the progression of the transmissible spongiform encephalopathies (TSEs), otherwise known as prion diseases [3]. These diseases are associated with the conversion of the normal cellular prion protein (PrP^C^) to an alternatively folded isoform (PrP^Sc^) [4]. The accumulation of PrP^Sc ^is closely associated with the main pathological features of TSEs: the spongiform degeneration of the brain, synaptic alterations, glial cell activation and extensive neuronal loss [5,6]. While a recent study reported that prion infection *in vivo *was associated with changes in brain cholesterol levels [7], the change in cholesterol regulation in neurons following prion infection has not been characterised extensively. Furthermore, because the brain is composed of diverse cell types, it is possible that changes in the cholesterol content of neurons may be obscured in mixed cell populations or whole brain studies. To reduce the problem of cell heterogeneity, the effects of prion infection on two neuronal cell lines were examined. We report that prion infection is associated with increased amounts of free cholesterol in the cell membrane, but also with reduced amounts of cholesterol esters suggesting that prion infection alters cholesterol regulation. The effects of prion infection on cholesterol balance were reproduced in primary cortical neurons incubated with exogenous PrP^Sc ^preparations.

Disturbing cholesterol metabolism within cells may have profound effects on cell function. Although cholesterol is a component of normal cell membranes, the amounts of free cholesterol are increased between three- and five-fold in specialised detergent-resistant micro-domains within the plasma membrane that are commonly called lipid rafts [8]. Such lipid rafts are also highly enriched in sphingolipids and gangliosides, and contain specific proteins [9]. The raft-associated proteins include many proteins attached to membranes via a glycosylphosphatidylinositol (GPI) anchor [10] including both PrP^C ^and PrP^Sc ^[11]. In addition, cellular receptors for folate or the p75 neurotrophin receptor are found within rafts [12,13], as are receptors for neurotransmitters including acetylcholine [14] and gamma-aminobutyric acid [15]. Such domains also contain components of signalling pathways including the Src family tyrosine kinases [16], adenylyl cyclase [17], the trimeric G-proteins [18] and cytoplasmic phospholipase A_2 _(cPLA_2_) [19]. Lipid rafts act as membrane platforms that concentrate molecules for cell signalling [20] and changes in membrane cholesterol levels may lead to abnormal cell signalling. As the neurotoxicity of PrP^Sc ^was blocked by PLA_2 _inhibitors [21] the effects of prion infection on PLA_2 _activity was examined. Here we report increased activation (phosphorylation) of cPLA_2 _in ScGT1 cells.

## Results

### Prion infection increased free cholesterol in neuronal cell lines

The amounts of protein and cholesterol in two prion-infected neuronal cell lines (ScN2a and ScGT1 cells) were compared to that of uninfected controls (N2a and GT1 cells). There were no significant differences in the amounts of protein between infected and uninfected cells. In contrast, the amounts of total cholesterol (a mixture of free and esterified cholesterol) were significantly higher in infected ScGT1 cells than in GT1 cells (542 ng cholesterol/mg protein ± 44 versus 453 ± 72, *n *= 11, *P *= 0.004) (Table 1). More detailed analysis showed that the amounts of free cholesterol within ScGT1 cells were 36% higher than those in GT1 cells (500 ± 54 versus 368 ± 59, *n *= 11, *P *= 0.0003), while the amounts of esterified cholesterol were 50% less than in GT1 cells (42 ± 14 versus 85 ± 28, *n = *11, *P = *0.0007). Similar results were obtained when ScN2a and N2a cells were compared: amounts of free cholesterol in ScN2a cells were 23% higher than in N2a cells (473 ± 41 versus 384 ± 37, *n = *11, *P = *0.0001), but the amounts of esterified cholesterol were significantly lower than those of N2a cells (52 ± 14 versus 87 ± 19, *n = *11, *P = *0.002). Thus, in both cell lines prion infection was associated with a significant decrease in the amounts of cholesterol esters and in the percentage of cholesterol that was esterified (Table 1).

**Table 1:** Prion infection increased the amounts of free cholesterol in neuronal cell lines. The amounts of total cholesterol, free cholesterol and esterified cholesterol (ng cholesterol/mg protein) in extracts from prion infected neuronal cells (ScN2a or ScGT1 cells) compared with their non-infected counterparts (N2a or GT1 cells). Values shown are the mean ± SD from 11 samples.

	**Cholesterol (ng/mg protein)**			
	
			**Esterified**	
			
	**Total (mean ± SD)**	**Free (mean ± SD)**	**Mean ± SD**	**Percentage**
**N2a**	472 ± 47	384 ± 37	87 ± 19	18 ± 4
**ScN2a**	525 ± 34	473 ± 41	52 ± 14	10 ± 3
**GT1**	453 ± 72	368 ± 59	85 ± 28	19 ± 5
**ScGT1**	542 ± 44	500 ± 54	42 ± 14	8 ± 3

**Figure 1:**
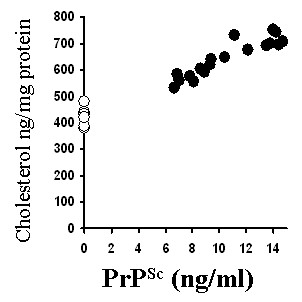
**Correlation between the amounts of PrP^Sc ^and free cholesterol in ScGT1 cells**. The amounts of PrP^Sc ^in GT1 cells (○) or ScGT1 cells (●) treated with varying amounts of GDNF for 7 days were plotted against the amounts of free cholesterol in the same cells (expressed as ng cholesterol/mg protein).

Brain-derived neurotrophic factor (BDNF) increased the PrP^Sc ^content of ScGT1 cells [22]. Here we report that treatment with BDNF, glial-derived neurotrophic factor (GDNF) or retinoic acid also increased the PrP^Sc ^content of ScGT1 cells, while treatment with nerve-growth factor (NGF) did not (Table 2). The increased PrP^Sc ^content of treated ScGT1 cells was accompanied by increased amounts of free cholesterol. The free cholesterol content of ScGT1 cells was significantly higher in cells treated with BDNF (654 ng cholesterol/mg protein ± 61 versus 510 ± 48, *n = *9, *P = *0.003), GDNF (655 ± 59 versus 510 ± 48, *n = *9, *P = *0.004) or retinoic acid (705 ± 83 versus 510 ± 48, *n = *9, *P = *0.002) but not in cells treated with NGF (503 ± 72 versus 510 ± 48, *n = *9, *P = *0.64). None of the treatments increased the cholesterol content of uninfected GT1 cells showing that the increases in free cholesterol were related to the PrP^Sc ^content of cells. To examine this relationship further, ScGT1 cells were treated with varying concentrations of GDNF and amounts of PrP^Sc ^and free cholesterol were measured. A significant correlation coefficient between the amounts of PrP^Sc ^and free cholesterol was observed (Pearson correlation = 0.922); see Figure 1.

**Table 2:** Correlation between PrP^Sc ^and free cholesterol content in ScGT1 cells. The amounts of PrP^Sc ^and free cholesterol in ScGT1 and GT1 cells treated for 7 days with neurotrophic factors or retinoic acid as shown. Free cholesterol was measured in cell extracts using the Amplex Red cholesterol assay kit. Values shown are the mean ± SD from 9 samples. PrP^Sc ^content of cells significantly greater than those of untreated cells (*P *< 0.05) are indicated by * and free cholesterol content of cells significantly greater than those of untreated cells (*P *< 0.05) are indicated by #.

	**PrP^Sc ^(ng/ml)**		**Free cholesterol (ng/mg protein)**	
	
	**ScGT1**	**GT1**	**ScGT1**	**GT1**
**Control**	6.9 ± 0.2	-	510 ± 48	392 ± 44
**10 ng/ml BDNF**	15.2 ± 1.4*	-	654 ± 61^#^	417 ± 43
**10 ng/ml GDNF**	14.2 ± 0.5*	-	655 ± 59^#^	393 ± 32
**10 ng/ml NGF**	7.4 ± 1.7	-	503 ± 72	364 ± 45
**100 nM retinoic acid**	16.5 ± 2.1*	-	705 ± 83^#^	440 ± 59

### PrP^Sc ^increases the free cholesterol content of cortical neurons

**Figure 2:**
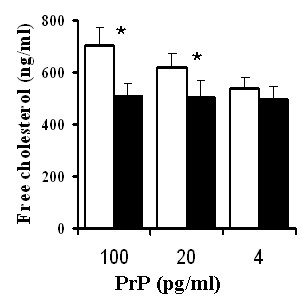
**PrP^Sc ^causes a dose-dependent increase in the free cholesterol concentration of cortical neurons**. The amounts of free cholesterol expressed as ng cholesterol/mg protein in cortical neurons treated for 48 hours with varying concentrations of PrP^C ^(■) or PrP^Sc ^(□). Amounts of free cholesterol significantly greater than those of untreated neurons (*P *< 0.05) are indicated with *.

As the above observations were on prion-infected neuronal cell lines, we sought to determine whether PrP^Sc ^had the same effect on non-transformed cells. Primary cortical neurons were incubated with sub-lethal amounts of PrP^Sc^, or equivalent amounts of PrP^C ^for 48 hours. The addition of PrP^Sc ^increased the amounts of free cholesterol when compared with untreated cells or cells treated with PrP^C ^(Figure 2). The amounts of free cholesterol were significantly higher in neurons treated with 100 pg/ml PrP^Sc ^than in untreated cells (704 ng/mg protein ± 73 versus 504 ± 58, *n = *9, *P = *0.0019). Similarly, the amounts of free cholesterol were significantly higher in neurons treated with 20 pg/ml PrP^Sc ^(632 ± 46 versus 504 ± 58, *n = *9, *P = *0.004). There was no significant difference in the amounts of free cholesterol in untreated neurons and in neurons incubated with 100 pg/ml PrP^C ^(504 ± 58 versus 517 ± 46, *n = *9, *P = *0.51).

### Stimulating cholesterol synthesis increases cholesterol esters but not free cholesterol in neurons

**Figure 3:**
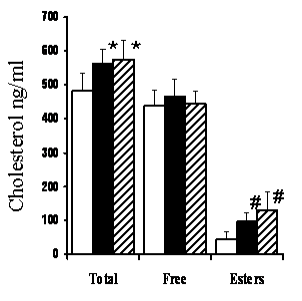
**Regulation of free cholesterol in cortical neurons**. The amounts of total cholesterol, free cholesterol and cholesterol esters, all expressed as ng cholesterol/mg protein in cortical neurones treated for 24 hours in control medium (open bars), 100 μM mevalonate (black bars) or 50 μM cholesterol (striped bars). Amounts of total cholesterol significantly greater than those of untreated neurons (*P *< 0.05) are indicated with * and amounts of cholesterol esters significantly greater than those of untreated neurons (*P *< 0.05) are indicated with #.

The possibility that prion infection stimulated cholesterol synthesis was examined by comparing the effects of PrP^Sc ^with those of increased cholesterol biosynthesis in cortical neurons. Mevalonate is a precursor of cholesterol synthesis that is a product of 3-hydroxy-3-methylglutaryl coenzyme A (HMG-CoA) reductase, the rate-limiting step in cholesterol synthesis [23]. Treatment with 100 μM mevalonate significantly increased the amounts of total cholesterol in neurons (562 ng cholesterol/mg protein ± 45 versus 482 ± 54, *n = *9, *P = *0.001); this increase consisted primarily of cholesterol esters (96 ± 25 versus 42 ± 24, *n = *9, *P = *0.006) as the amounts of free cholesterol were unchanged (466 ± 50 versus 440 ± 46, *n = *9, *P = *0.16). Similar results were obtained in cells treated with 10 μM cholesterol, which increased the amounts of cholesterol esters (130 ± 55 versus 42 ± 24, *n = *9, *P = *0.001) but not free cholesterol (446 ± 36 versus 440 ± 46, *n = *9, *P = *0.54); see Figure 3. The percentage of cholesterol that was esterified in untreated cells (9% ± 4) was raised to 17% ± 5 in neurons incubated with mevalonate, and 22% ± 8 in neurons treated with cholesterol. The addition of mevalonate or cholesterol did not affect the protein content of cell extracts.

### Prion infection increased activation of cPLA_2 _in neuronal cell lines

**Figure 4:**
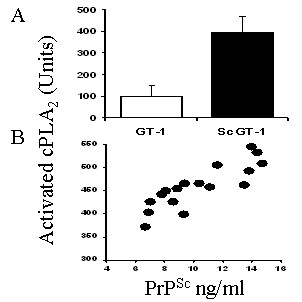
**Prion infection increases the amounts of activated cPLA_2 _in ScGT1 cells**. (A) The amounts of activated cPLA_2 _in cell extracts from 1 × 10^6 ^GT1 cells (open bars) or ScGT1 cells (black bars). Values shown are the mean ± SD from 10 samples. (B) The amounts of activated cPLA_2 _in ScGT1 cells treated with varying concentrations of GDNF for 7 days (●) were plotted against the amounts of PrP^Sc ^in the same cells.

As lipid rafts act as platforms in which signalling complexes assemble [24], the possibility that the altered composition of lipid rafts in prion-infected cells affected cell signalling was examined. More specifically, as PLA_2 _was required for prion formation [25], the amounts of activated cPLA_2 _in cells were examined. The amounts of activated (phosphorylated) cPLA_2 _in ScGT1 cells were greater than those in GT1 cells (Figure 4A). The relationship between activated cPLA_2 _and PrP^Sc ^was examined in ScGT1 cells treated with different neurotrophins or retinoic acid. The increased PrP^Sc ^content of treated ScGT1 cells was accompanied by increased amounts of activated cPLA_2_. The amounts of activated cPLA_2 _in ScGT1 cells was significantly higher in cells treated with BDNF (514 units/ml ± 69 versus 395 ± 33, *n = *9, *P = *0.01), GDNF (542 ± 65 versus 395 ± 33, *n = *9, *P = *0.008) or retinoic acid (553 ± 79 versus 395 ± 33, *n = *9, *P = *0.01) but not in cells treated with NGF (416 ± 46 versus 395 ± 33, *n = *9, *P = *0.41). In contrast, none of these treatments significantly increased the amounts of activated cPLA_2 _in GT1 cells showing that increased activation of cPLA_2 _is related to the PrP^Sc ^content of cells (Table 3). The relationship between cPLA_2 _and PrP^Sc ^was examined further in ScGT1 cells treated with varying concentrations of GDNF. The correlation coefficient between the amounts of PrP^Sc ^in cells and the amounts of activated cPLA_2 _was significant (Pearson correlation = 0.865); see Figure 4B.

**Table 3:** Correlation between PrP^Sc ^and activated cPLA_2 _in ScGT1 cells. The amounts of PrP^Sc ^and activated cPLA_2 _(phosphorylated at serine 505) in ScGT1 and GT1 cells treated for 7 days with neurotrophic factors or retinoic acid as shown. Values shown are the mean ± SD from 9 samples. PrP^Sc ^content of cells significantly greater than those of untreated cells (*P *< 0.05) are indicated by * and amounts of activated cPLA_2 _significantly greater than those of untreated cells (*P *< 0.05) are indicated by #.

	**PrP^Sc ^(ng/ml)**		**Activated cPLA_2 _(units)**	
	**ScGT1**	**GT1**	**ScGT1**	**GT1**

**Control**	6.9 ± 0.2	-	395 ± 33	100 ± 18
**10 ng/ml BDNF**	15.2 ± 1.4*	-	514 ± 69^#^	102 ± 7
**10 ng/ml GDNF**	14.2 ± 0.5*	-	542 ± 65^#^	109 ± 9
**10 ng/ml NGF**	7.4 ± 1.7	-	416 ± 46	98 ± 6
**100 nM retinoic acid**	16.5 ± 2.1*	-	553 ± 79^#^	113 ± 13

Confirmation of increased PLA_2 _activity in ScGT1 cells was provided by observations that the amounts of PGE_2 _produced by ScGT1 cells were significantly higher than that of GT1 cells (318 pg/ml PGE_2 _± 46 versus 134 ± 37, *n = *6, *P = *0.000004). Similarly, the amounts of PGE_2 _produced by ScN2a cells were significantly higher than that of N2a cells (253 ± 40 versus 118 ± 13, *n = *6, *P = *0.0003). To ensure that PGE_2 _was produced via the PLA_2_/cyclo-oxygenase pathway, ScGT1 cells were treated with PLA_2 _or cyclo-oxygenase inhibitors. The addition of the PLA_2 _inhibitors reduced PGE_2 _production in ScGT1 cells; treatment with 1 μM AACOCF_3 _reduced PGE_2 _levels from 318 ± 46 to 98 ± 28, *n = *6, *P = *0.000002, while 1 μg/ml aristolochic acid reduced PGE_2 _production to 124 ± 36, *n = *6, *P = *0.00001. Cyclo-oxygenase inhibitors also reduced PGE_2 _production, 100 nM acetyl-salicylic acid reduced PGE_2 _levels from 318 ± 46 to 59 ± 34, *n = *6, *P = *0.0000005, and 500 nM ibuprofen reduced PGE_2 _production to 110 ± 40, *n = *6, *P = *0.00007.

### Rafts containing PrP^Sc ^also contain cPLA_2_

**Figure 5:**
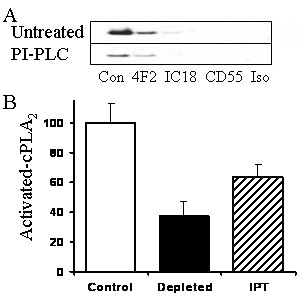
**Activated cPLA_2 _is associated with PrP^Sc ^in ScGT1 cells**. (A) Immunoblots showing amounts of cPLA_2 _immunoprecipitated with antibodies to PrP (4F2 or IC18), CD55 or an isotype control (Iso) from untreated ScGT1 cells (Untreated) or from ScGT1 cells pre-treated with 0.2 units PI-PLC for 30 minutes (PI-PLC). (B) The amounts of activated cPLA_2 _(expressed as a percentage) in whole cell extracts from ScGT1 cells (open bars), from ScGT1 cell extracts depleted with the anti-PrP mab 4F2 (black bars) and in 4F2 immunoprecipitates (IPT) from ScGT1 cell extracts (striped bars). Values shown are the mean ± SD from 10 samples.

Immunoprecipitation was used to determine whether cPLA_2 _was associated with PrP-containing lipid rafts in ScGT1 cells. Mab 4F2, which recognises both PrP^C ^and PrP^Sc^, precipitated cPLA_2 _out of membrane extracts from untreated ScGT1 cells. Two methods were used to show that cPLA_2 _was associated with PrP^Sc ^rather than PrP^C ^in these ScGT1 cell extracts. First, immunoprecipitation with mab IC18, which recognises PrP^C ^but not PrP^Sc^, did not precipitate cPLA_2 _from ScGT1 cells. Second, immunoprecipitation with mab 4F2 precipitated cPLA_2 _from ScGT1 cells from which PrP^C ^had been removed following treatment with PI-PLC (Figure 5A). A mab to CD55, or an IgG_2 _isotype control, did not precipitate cPLA_2 _from ScGT1 cells. Next we examined the distribution of activated cPLA_2 _within ScGT1 cells. The amounts of activated cPLA_2 _in whole cell extracts (100%) were compared with those in membranes precipitated with mab 4F2 and to the depleted membrane extract. Greater than 60% of activated cPLA_2 _was found in the immunoprecipitated membrane fraction (Figure 5B).

## Discussion

The major goal of this study was to investigate the impact of PrP^Sc ^on the biochemistry of cell membranes. The amounts of total cholesterol in membranes were significantly higher in prion-infected cell lines than in their uninfected counterparts and there was a significant correlation between amounts of cholesterol and PrP^Sc^. More specifically, prion infection was associated with a significant increase in the amounts of free cholesterol. While much of what is known about the role of cholesterol in cell membranes is surmised from the changes in cells brought about by cholesterol depletion, either from cholesterol synthesis inhibitors or via cholesterol extraction, little is known about how neurons respond when the cholesterol content of membranes is increased.

How the presence of prions affects cholesterol levels remains to be determined. A synthetic prion-derived peptide activated HMG-CoA reductase suggesting a mechanism by which prion infection increased cholesterol production [26]. However, we were unable to replicate the effects of prion infection in non-infected cells by stimulating cholesterol biosynthesis or by adding exogenous cholesterol. The addition of mevalonate or cholesterol did not increase the amounts of free cholesterol in cell membranes; rather, they increased the amounts of cholesterol esters. This contrasts with the situation in prion-infected cells where the amounts of cholesterol esters were reduced. Cholesterol in cells is found either as free cholesterol in membranes or as cholesterol esters in cytoplasmic droplets. A dynamic equilibrium between the pools of free cholesterol and cholesterol esters is tightly controlled by acyl-coenzyme A:cholesterol acyltransferase (ACAT), an endoplasmic reticulum (ER)-resident enzyme that catalyses the formation of cholesterol esters from cholesterol and long-chain fatty acids [27]. In uninfected cells excess free cholesterol activates ACAT resulting in increased production of cholesterol esters. In these cells, increased free cholesterol levels were only seen following the addition of a combination of free cholesterol and an ACAT inhibitor (data not shown).

The situation in prion-infected cells, where the increased amounts of free cholesterol is accompanied by reduced amounts of cholesterol esters, is unusual. The increase in free cholesterol and the reduction of cholesterol esters in prion-infected cells may be a result of direct inhibition of ACAT or by sequestration of cholesterol in micro-environments that avoid ACAT. The amount of cholesterol in cell membranes is partly determined by its fatty acid composition. The high incidence of saturated fatty acids attached to sphingolipids, gangliosides and GPI-anchored proteins allows tight molecular packing and increases the solubilisation of free cholesterol [28]. Thus, the formation of PrP^Sc ^may have a direct effect on the composition of cell membranes as the self-aggregation of PrP^Sc ^results in the clustering of GPI anchors attached to PrP^Sc^. The increased density of saturated fatty acids within PrP^Sc^-containing micro-domains encourages the solubilisation of free cholesterol and the remodelling of those membranes.

Increasing the free cholesterol content of membranes is thought to reduce membrane fluidity and subsequently affect the endocytosis and trafficking of proteins. Therefore, the formation of PrP^Sc ^may alter conventional lipid raft structure and the PrP^C^-protein interactions that occur within lipid rafts. For example, PrP^C ^has been reported to bind to caveolin-1 [29] or N-CAM [30], proteins that reside within lipid rafts. It is unclear whether these protein-protein interactions are affected following the conversion of PrP^C ^to PrP^Sc^. The sequestration of free cholesterol into PrP^Sc^-containing lipid rafts may deplete free cholesterol from other cellular pools where it helps to stabilise the packing of sphingolipids, gangliosides and raft-associated proteins in the membrane. This may affect the function of such proteins. For example, free cholesterol affects the formation and function of synapses [31]. Therefore, sequestration of cholesterol by PrP^Sc ^may affect synaptic transmission, a hypothesis supported by observations that ScGT1 cells contain altered amounts of synaptic proteins including synaptophysin [32] and that synapse damage is seen during the early stages of experimental prion diseases [6].

Cholesterol-dependent micro-domains are increasingly implicated as platforms necessary for cell signalling [24] and scrapie infections of neuronal cells are associated with increased levels of Src kinase [33]. The activation of PLA_2 _that is necessary for prion formation [25] is reduced in cholesterol-depleted cells suggesting that this enzyme may reside within a lipid raft [34]. Here we show that ScGT1 cells contained four times as much activated cPLA_2 _as GT1 cells and there was a significant correlation between amounts of activated cPLA_2 _and PrP^Sc^. Immunoprecipitation studies showed that activated cPLA_2_co-localised with PrP^Sc^-containing lipid rafts in ScGT1 cells. Previous studies showed that cell activation results in the translocation of cPLA_2 _to endoplasmic and plasma membranes [35]. Our observations are consistent with the hypothesis that prion infection stimulates the translocation of cPLA_2 _to lipid rafts containing PrP^Sc^. The activation of cPLA_2 _is associated with the production of prostaglandins and the amounts of PGE_2 _produced by ScGT1 cells were significantly higher than that of GT1 cells. Our observation that pre-treatment of ScGT1 cells with PLA_2 _or cyclo-oxygenase inhibitors reduced PGE_2 _production showed that PGE_2 _was a valid measure of PLA_2 _activity in these cells. These findings are consistent with reports of increased PGE_2 _in murine scrapie [36] and raised levels of PGE_2 _in the cerebrospinal fluid of patients with Creutzfeldt-Jakob disease [37].

## Conclusion

We have demonstrated that the presence of PrP^Sc ^increased the free cholesterol content of cell membranes. The increased free cholesterol could not be replicated by the stimulation of cholesterol synthesis or by the addition of exogenous free cholesterol, which increased the amounts of cholesterol esters. Our observations are consistent with the hypothesis that the clustering of saturated fatty acids, parts of the GPI anchors attached to PrP^Sc^, increased the amounts of free cholesterol solubilised within the plasma membrane which increased membrane rigidity. These changes in cell membranes could reduce endocytosis and the recycling of cholesterol through the ER where it is exposed to ACAT, consistent with reduced cholesterol ester production in infected cells. The increased amounts of free cholesterol in the plasma membrane were associated with increased activation of the PLA_2 _pathway that is necessary for PrP^Sc^-mediated neurotoxicity. This is a rare example of an infective agent increasing free cholesterol levels within cell membranes and raises the possibility that disturbances in membrane cholesterol are major triggering events in neurodegenerative diseases.

## Methods

### Cell lines

Prion-infected ScGT1 cells, from a murine hypothalamic neuronal cell line infected by the Chandler scrapie isolate and ScN2a neuroblastoma cells, were grown in Hams F12 medium supplemented with 2 mM glutamine, 2% foetal calf serum (FCS) and standard antibiotics (100 U/ml penicillin and 100 μg/ml streptomycin; Invitrogen, Paisley, UK). Uninfected N2a or GT1 cells were used as non-infected controls and grown in the same medium. To determine the effect of neurotrophins or retinoic acid, cells were plated at 1 × 10^5 ^cells/well in 6 well plates. Cells were then grown with daily changes of media for 7 days.

### Neuronal cultures

Primary cortical neurons were prepared from the brains of mouse embryos (day 15.5) after mechanical dissociation, cell sieving and isolation on histopaque (Sigma). Neuronal precursors were plated (1,000,000 cells/well in 24 well plates coated with 5 μg/ml poly-L-lysine) in Hams F12 containing 5% FCS for 2 hours. Cultures were shaken (600 rpm for 5 minutes) and non-adherent cells removed by two washes in phosphate buffered saline (PBS). Neurons were grown in neurobasal medium (NBM) containing B27 components (Invitrogen) for 7 days and subsequently incubated with test compounds. Immunolabelling studies showed that after 7 days cultures contained less than 5% glial cells (about 3% GFAP positive and less than 1% MAC-1 positive cells).

### Cell extracts

At the end of the treatment, cells were washed twice in PBS and homogenised in an extraction buffer containing 10 mM Tris-HCl, 100 mM NaCl, 10 mM EDTA, 0.5% Nonidet P-40, 0.5% sodium deoxycholate and 0.2% sodium dodecyl sulphate (SDS) at 1 × 10^6 ^cells/ml. Mixed protease inhibitors (AEBSF, Aprotinin, Leupeptin, Bestain, Pepstatin A and E-46; from Sigma) were added to some cell extracts. Membranes were prepared by repeated passage with a Wheaton homogeniser; nuclei and large fragments were removed by centrifugation (300 × *g *for 5 minutes). To determine the amount of PrP^Sc ^in cells these supernatants were digested with 1 μg/ml proteinase K for 1 hour at 37°C, digestion was stopped with mixed protease inhibitors. The soluble material was heated to 95°C for 5 minutes and tested in a PrP specific enzyme-linked immunosorbent assay (ELISA).

### PrP ELISA

The amount of PrP present in cell extracts was determined in a sandwich ELISA using a capture mab (ICSM18 which recognises amino acids 146 to 159 of murine PrP). Samples were applied and detected with biotinylated mab ICSM35 (which recognises a region between amino acids 91 and 110). Biotinylated mab was detected using extravidin-alkaline phosphatase and 1 mg/ml 4-nitrophenyl phosphate in a diethanolamine buffer (Sigma). Absorbance was measured on a microplate reader at 450 nm and the amount of PrP in cell extracts was calculated by reference to a standard curve of recombinant murine PrP (Prionics, Zurich, Switzerland); its limit of detection was 0.05 ng/ml.

### Cholesterol and protein content

Cellular cholesterol and protein content were determined in cell extracts (1 × 10^6 ^cells/ml). Protein concentrations were measured using a micro-BCA protein assay kit (Pierce, Cramlington, UK). The amounts of cholesterol were measured using the Amplex Red cholesterol assay kit (Invitrogen), according to the manufacturer's instructions. Briefly, cholesterol is oxidised by cholesterol oxidase to yield hydrogen peroxide and ketones. The hydrogen peroxide reacts with 10-acetyl-3, 7-dihydroxyphenoxazine (Amplex Red reagent) to produce highly fluorescent resorufin, which is measured by excitation at 550 nm and emission detection at 590 nm. By performing the assay in the presence or absence of cholesterol esterase (50 units/ml) the assay can also determine the amounts of esterified cholesterol within samples.

### Isolation of PrP^Sc^/PrP^C^

PrP^C ^was extracted from uninfected GT1 cells by immunoprecipitation with a PrP-specific mab 4F2 and magnetic protein G beads followed by reverse-phase chromatography on a C18 column and a gradient of acetonitrile in water and 0.1% triflouroacetic acid. PrP^Sc ^was extracted from ScGT1 cells. Infectious cell extracts were digested with 1 μg/ml proteinase K (37°C, 1 hour) and PrP^Sc ^was precipitated with mab 4F2 and magnetic protein G beads. PrP^Sc ^was subsequently isolated by reverse-phase chromatography as above. The amounts of PrP^Sc^/PrP^C ^in different fractions were quantified by a PrP-specific ELISA.

### cPLA_2 _ELISA

The activation of cPLA_2 _is accompanied by phosphorylation of the 505 serine residue, which can be measured by phospho-specific antibodies. The amounts of activated cPLA_2 _in cell extracts were measured by a sandwich ELISA. Nunc Maxisorb immunoplates were coated with 0.5 μg/ml of mouse mab anti-cPLA_2_, clone CH-7 (Upstate, Milton Keynes, UK) in carbonate buffer for 1 hour and blocked with 10% FCS. Samples were incubated for 1 hour at room temperature and the amounts of activated cPLA_2 _were detected using a rabbit polyclonal anti-phospho-cPLA_2 _(Cell Signalling Technology). Bound antibodies were detected with biotinylated anti-rabbit IgG (Dako, Ely, UK), extravidin-alkaline phosphatase and 1 mg/ml 4-nitrophenyl phosphate in a diethanolamine buffer. Absorbance was measured at 450 nm and the amounts of activated cPLA_2 _were calculated from a standard curve using nonlinear regression. Samples were expressed as 'units cPLA_2_' where a measure of 100 units was defined as the amount of cPLA_2 _in 1 × 10^6^ untreated GT1 cells. A standard curve was generated from this sample using sequential log 2 dilutions (range 100 to 1.56 units/well).

### PGE_2 _assay

The amounts of PGE_2 _produced by cells were determined by using an enzyme-immunoassay kit (Amersham Biotech, Amersham, UK) according to the manufacturer's instructions. This assay is based on competition between unlabelled PGE_2 _in the sample and a fixed amount of labelled PGE_2 _for a PGE_2 _specific antibody. The detection limit of this assay is 20 pg/ml.

### Immunoprecipitations

Cells were washed in ice-cold PBS and incubated with antibodies against decay accelerating factor (DAF; CD55, mab BRIC 216, Bio-Products, Elstree, UK), PrP (mabs 4F2, ICSM18) or an isotype control in ice-cold PBS containing 5% FCS. In some experiments ScGT1 cells were pre-treated with 0.2 units/ml PI-PLC for 1 hour prior to immunoprecipitation to remove surface PrP^C^. After 30 minutes cells were washed 5 times in ice-cold PBS and solubilised with 1% Triton × 100, 10 mM Tris-HCl, 100 mM NaCl, 10 mM EDTA and protease inhibitors at 1 × 10^6 ^cells/ml for 1 hour at 4°C (this detergent solubilises the normal cell membrane but does not affect lipid raft micro-domains). Cell debris was removed from membrane preparations by centrifugation (300 × *g *for 5 minutes) and the supernatant was incubated with μMACS protein G microbeads (10 μl/ml; Miltenyi Biotech, Bisley, UK) for 30 minutes. Protein G bound antibody complexes were isolated using a μMACS magnetic system and standard protocols (Miltenyi Biotech). For ELISA studies proteins were eluted in 10 mM Tris-HCL, 150 mM NaCl, 10 mM EDTA, 0.5% Nonidet P-40, 0.5% sodium deoxycholate, 0.2% SDS and mixed protease inhibitors at 1 × 10^6 ^cells/ml. Other beads were boiled for 5 minutes in Laemmli buffer (Bio-Rad) and subjected to electrophoresis on a 15% polyacrylamide gel. Proteins were transferred onto a Hybond-P PVDF membrane (Amersham Biotech, UK) by semi-dry blotting. Membranes were blocked using 10% milk powder and cPLA_2 _was detected by mab CH-7. Bound antibody was detected by a secondary anti-mouse IgG conjugated to peroxidise and an enhanced chemiluminescence kit (Amersham Biotech).

### Reagents

Mevalonate and AACOCF_3 _were bought from Calbiochem, Nottingham, UK. Acetyl salicylic acid, ibuprofen, MAPF, cholesterol, retinoic acid, BDNF, GDNF and NGF were obtained from Sigma.

### Statistical analysis

Comparison of treatment effects was carried out using one- and two-way analysis of variance techniques as appropriate. *Post hoc *comparisons of means were performed as necessary. For all statistical tests significance was set at the 5% level.

## Competing interests

The author(s) declare that they have no competing interests.

## Authors' contributions

CB was responsible for the conception, planning and performance of experiments and for writing this manuscript. Both MT and AW contributed to the planning of experiments, interpretation of results and the writing of the manuscript.
